# Analysis of Novel Porosity–Water-to-Binder Index for Prediction of Strength, Stiffness and Durability for Cemented Soils

**DOI:** 10.3390/ma16196354

**Published:** 2023-09-22

**Authors:** Jair Arrieta Baldovino, Yamid E. Nuñez de la Rosa, Oriana Palma Calabokis

**Affiliations:** 1 Civil Engineering Program, Universidad de Cartagena, Cartagena de Indias 130015, Colombia; 2 Faculty of Engineering and Basic Sciences, Fundación Universitaria Los Libertadores, Bogotá 1112211, Colombia; yenunezd@libertadores.edu.co (Y.E.N.d.l.R.); opalmac@libertadores.edu.co (O.P.C.)

**Keywords:** porosity–water/binder index, unconfined compressive, split tensile, stiffness, durability

## Abstract

The initial weight and volume relationships are crucial factors in determining the strength, stiffness, and durability of cement-stabilized soils. The porosity/binder ratio has been widely used as a control parameter for stabilized soil mixtures. However, this approach does not take into consideration the water content used during the stabilization process, which can impact the strength and durability of the final product. To address this issue, this paper introduces the porosity–water/binder relationship as a new parameter to predict the strength, stiffness (*G_o_*), and durability against wetting–drying cycles of artificially cemented soils. The strengths, *G_o_*, and accumulated losses of mass (*ALM*) of different stabilized soils were compared based on this new parameter, and the comprehensive results were analyzed to demonstrate its effectiveness. The findings indicate that the new parameter is a suitable design parameter for soil–lime, soil–cement, and geopolymerized soil mixtures. Furthermore, it was determined that the water content has no effect on the splitting tensile strength to compressive strength ratio. The results of this study offer valuable insights into the optimization of stabilized soils and the development of improved soil stabilization processes.

## 1. Introduction

The initial weight–volume relationships are known to be factors that control the general mechanical behavior of cement-improved soils, regardless of the soil grain size and cementitious agent applied (e.g., conventional Portland cement, lime, alkali-activated cement). These relationships include the relationships between parameters such as soil volume, porosity, binder volume, void ratio, and degree of saturation. In addition to lime or cement, other stabilizing agents can be added, such as construction and demolition waste, biomass ash, fibers, and even different granulometric soils [1,2]. The use of alternative materials and industrial by-products in artificially cemented soils has also gained considerable attention and practical applicability, according to Hanafi et al. [3]. In this context, different studies have applied waste-like materials, such as grounded fiberglass, fly ash, and rice husk ash, as artificial cementation materials [4]. Therefore, currently, the use of artificial cementation in soils as improvement agents is attracting significant industrial interest. However, the dosing methods available for this technique are time consuming and require several tests before application. Other studies applied semi-empirical models for predicting the mechanical properties of geomaterials. Ermolovich et al. [5] examines losses in the development of low-value water-soluble ore deposits and emphasizes the importance of utilizing backfill systems. They propose using industrial waste from water-soluble ore processing to replace specially produced inert components in backfill preparation. The experimental results show that adding astralene as a nanomodifying additive to the backfill increases its strength properties by 1.76–2.36 times while reducing binder consumption. The study concludes that using nanomodified backfill based on water-soluble ore enrichment waste offers economic and ecological benefits by reducing losses, decreasing waste storage costs, and improving mining operation safety. In addition, Kanty et al. [6] presents laboratory testing of organic soil–cement samples, concluding that Deep Soil Mixing (DSM) may not be suitable for organic soils due to risks related to settlement and material capacity. The study recommends core drilling for assessing material mixing and strength when designing DSM columns in organic soils.

The dosing method that considers the relationship between porosity (*η*) and volumetric cement content (*C_iv_*), which are key factors in determining soil strength, was established by Consoli et al. [7] based on a study of the most impactful variables. This index was proposed as a rational method to achieve the desired strength by modifying the ratio (*η*/*C_iv_*) between the porosity and the cement content to be added. Consoli et al. [7] demonstrated the construction of a characteristic curve that can be used to achieve different soil strengths. This curve, which is defined according to the type of material, plots the evolution of the strength (*q_u_*) with respect to the variation in the voids or the porosity/cement ratio. Since then, the *η*/*C_iv_* index has been extended to soils stabilized with lime (called the porosity/lime index). Despite major improvements in dosing techniques, the field of pozzolan-based treatments for soil stabilization has not seen much progress. Most pozzolans do not exhibit cementitious properties and require activators for cementing. Based on this requirement, the *η*/*B_iv_* (porosity/binder) ratio was calculated. This new method focuses on the ratio between the porosity and the total amount of volumetric binder used. The latter corresponds to the sum of the absolute volumes of the lime/cement and pozzolan. In general, in agreement with the results of several studies [8,9], an increase in the *η*/*B_iv_* index results in a reduction in the unconfined compressive strength. This effect is caused by an increase in porosity, which decreases the interlocking of particles, thereby resulting in a reduction in the frictional strength and/or chemical cementation caused by the reduced availability of agglomerating agents for their development.

Regardless of the relationship applied, the authors verified the need to use an exponent *β* (e.g., *η*/*B_iv_^β^*) that would increase the compatibility of the proposed relationships. Therefore, in composites where the porosity has a greater impact, the exponent *β* in the ratio is less than 1. Otherwise, it is greater than 1, indicating that cement reactions play a more important role. Furthermore, the index was not only theoretically derived, but studies have also proven that its formulations are not solely based on empirical evidence. Studies performed by Diambra et al. [10,11] on soils stabilized with cement, as well as the research conducted by Festugato et al. [12] on soils improved using fibers along with cement, have indicated the possibility of arranging both the empirical and theoretical equations based on the concept of combining the strength contributions of the mixture of materials. These equations were designed to describe the unconfined compressive and tensile strengths of soils treated with cement, as shown below.

(1) $$q_{u}=A{\left[\frac{\eta }{{\left(C_{iv}\right)}^{x}}\right]}^{-C}$$

The constant A in the Equation (1) is of significant theoretical importance, as it depends on various factors that influence the mechanical behavior of the soil–cement mixture. These factors include the soil’s critical state strength ratio, the cement’s uniaxial compressive strength, the cement stress ratio, the porosity at a critical state, and the ratio between the uniaxial compression and extension strengths [10]. The exponents -*C* and *x* play a vital role in characterizing the relationship between the soil and cement properties and the strength behavior of the mixture.

The values of *x* and *C* in this equation depend on the soil matrix. The parameter *A*, which is a multiplying factor, is the outcome of the combination of the soil matrix properties, cement phase properties, and curing time. Furthermore, Diambra et al. [10,11] (and Festugato et al. [12]) stated that *A* is predominantly determined based on the frictional strength of the matrix and the strength of the cemented phase. Additionally, the exponent *C* depends on the properties of the soil matrix, which influences *A*.

Although a clear applicability of *η*/*B_iv_^b^* was observed in multiple studies [13,14,15], the index fails to consider the water content used during the soil stabilization process. Therefore, this study introduces the porosity–water/binder relationship as a new index that controls the splitting tensile (*q_t_*), unconfined compressive strength (*q_u_*), stiffness (*G*_o_), and accumulated loss of mass (*ALM*) of cemented soils. This study also aimed to create equations that allow for the calculation of the initial weight and volume relationships for various soil–binder mixture conditions. The binder content, dry weight at molding, and specific gravity of both the soil and binders are unique parameters for developing the equations. Finally, the relationships between the initial weight and molding volume were used to verify their impacts on the mechanical behavior of soil–binder mixtures using a unique parameter called porosity–water/binder. Several types of artificially cemented soils were used to study this effect. 

## 2. Methodology

The methodology was divided into 3 parts. The first part consists of the theoretical development of the porosity-to-cement index. The second part consists of the semi-empirical development of a new porosity–water/binder index where the amount of water in volume is introduced. The last part consists of an experimental survey of studies on artificially cemented soils where the initial relationship (i.e., porosity–binder) has been used to apply the proposed relationship and thus demonstrate its efficiency to predict strength, stiffness, and durability properties.

The fundamental properties of interest to be enhanced when implementing the soil–cement technique are those regarding strength, deformability, and durability. These properties are directly influenced by the conditions under which the treatments are carried out. Therefore, it is essential to comprehend how this process impacts the soil’s characteristics. Lade and Trads [16] point out that the three most impactful factors influencing the stress–strain response and volumetric behavior of artificially cemented granular soils are effective confining pressure, initial void ratio, and cement content. In addition to these, other factors related to the physicochemical characteristics of the soil are also significant, such as soil texture and moisture content during compaction, as they affect the alteration of soil–cement mixture behavior, regardless of the soil’s inherent nature.

### 2.1. Theoretical Derivation of Porosity-to-Cement Index

Diambra et al. [10] have assumed that the artificially cemented soil is an isotropic composite material in which the failure is determined by superimposing the strength contributions of either the cement or the soil phases. Moreover, it assumed the strain compatibility between these two phases and, as well, it assumed a simultaneous failure in both. The strength of the cement phase was described by the Drucker–Prager failure criterion, whereas the resistance of the soil matrix was represented by relating the mean stress ratio to a state parameter in terms of the material’s porosity (current porosity/porosity at the critical state). The following relationship thus results from the aforementioned assumptions (Equation (2)):

(2) $$q_{u}=\frac{6\mu_{c}\sigma_{c}^{c}}{K_{c}\left(1-\beta \right)+3\left(\beta +1\right)}\left[\frac{K_{c}-M{\left(\frac{\eta_{cs}}{\eta }\right)}^{a}}{3-M{\left(\frac{\eta_{cs}}{\eta }\right)}^{a}}\right]$$

where $\mu_{c}=C_{iv}/100$. M is the critical state strength ratio of the soil; $\eta_{cs}$ is the critical state porosity (which is taken as a constant for each soil); *a* is the parameter governing the dependence of soil strength on its density; $\sigma_{c}^{c}$ is the cement phase compressive strength at a specified curing period; β is the uniaxial compression and extension cement strength ratio; and $K_{c}$ is the cement stress ratio.

Equation (2) does not resemble the empirical formula stated in Equation (1), as it is not a linear function of the peak strength of the soil. Yet, the following approximation can be introduced (Equation (3)):

(3) $$\frac{K_{c}-M^{*}}{3-M^{*}}≅M^{*}(-0.6+0.45K_{c})$$

If Equation (3) is introduced in Equation (2) with further manipulation, a full conformity between the theoretical and the empirical approaches can be reached, as shown in Equation (4). Through this approach, the coefficients *x* and *C* from Equation (1) on the soil matrix properties can be determined. Moreover, the scalar parameter *B* is the first term in the left-sided equation and is either influenced by the soil or the cement phase properties.

(4) $$\frac{6M\sigma_{c}^{c}(-0.6+0.45K_{c})\eta_{cs}^{a}}{100\left[K_{c}\left(1-\beta \right)+3(\beta +1)\right]}{\left[\frac{\eta }{{\left(C_{iv}\right)}^{1/a}}\right]}^{-a}=B{\left[\frac{\eta }{{\left(C_{iv}\right)}^{1/a}}\right]}^{-a}$$

To verify the effectiveness of Equation (4), Table 1 summarizes the experimental values of *a* and 1/*a* in different types of soil mixes with stabilizers (mainly cement). Theoretically, the value of “*p*” should be 1. It is notable that almost all experimental valleys have a value of 1, making Equation (4) effective, but without considering the volumetric quantity of water.

### 2.2. Semi-Empirical Derivation of the Novel Porosity–Water-to-Cement Index

The porosity/binder index (*η*/*B_iv_*) introduced by Consoli et al. [7] is a crucial tool for evaluating the unconfined compressive strength of artificially cemented soils. *B_iv_* is a ratio that is calculated by dividing the volume of the cementing agent by the total volume of the specimen, and it is expressed as a dimensionless value. The unconfined compressive strength (*q_u_*) and split tensile strength (*q_t_*) are functions of *η*/*B_iv_* adjusted to two empirical exponents (*x* and *C*) and an empirical constant *A_q_* (in kPa), as shown below:

(5) $$q_{u}\vee q_{t}=A_{q}{\left[\frac{\eta }{{\left(B_{iv}\right)}^{x}}\right]}^{-C}$$

The scalar *A_q_* is governed by the properties of both the soil and cementitious matrix. Therefore, Equation (5) is expanded to investigate the strength, stiffness, and durability of different mixtures that are stabilized using both traditional binders and geopolymers. One of the limitations of using this parameter is that it does not consider the volume of water within the mixture. Another disadvantage is that the range in which it can be used is unknown, because the strength tends to be infinite for minimal values. The value of *η*/*B_iv_* can be multiplied by the *C_w_*/*B_iv_* index, where *C_w_* is the volume of water divided by the total volume of the specimen. Thus, Equation (5) becomes:

(6) $$q_{u}\vee q_{t}=A{\left[\frac{\eta C_{w}}{{\left(B_{iv}\right)}^{2a}}\right]}^{-d}$$

where *a* and *d* are empirical parameters that depend on the soil and binder properties. This factor, called porosity–water/binder, can be introduced in other studies. Several authors [22] have already demonstrated that the cemented structure and arrangement between the grains are the main factors that govern the behavior of structured material, at least until a stress state is reached in which the plasticization phenomenon occurs. For cemented or structured soils, the plasticization phenomenon is directly related to the breakage of the cemented structure from irreversible plastic deformations, which causes a significant reduction in the strength and stiffness of the material, resulting in a visible discontinuity in the stress–strain behavior. Thus, volumetric water content plays a fundamental role in this process and must be considered [23]. Having a straightforward relationship for predicting the strength over time based on the water–cement and binder content would reduce the need for multiple tests to determine the optimal binder amount.

## 3. Results

In this section, the application of methodology of rational dosing through the porosity–water index/volumetric content of cementing agent (Equation (6)) is discussed and validated for the test results of: unconfined compressive strength (*q_u_*), split tensile strength (*q_t_*), ultrasonic pulse (*G_o_*), and durability with the evaluation of the accumulated loss of mass (*ALM*). Thus, such response variables were related to the porosity–water/binder index (*ηC_w_*/*B_iv_*) for all mixtures detailed in Table 2 and Table 3.

Table 2 presents the geotechnical properties of the soils used to study the effects of the new porosity–water/binder (*ηC_w_*/*B_iv_*) index on strength, durability, and stiffness. The properties include granulometric composition, Atterberg limits, specific gravity *G*s, mineralogy, and Unified Soil Classification System (USCS). Additionally, Table 3 presents, in detail, the types of mixtures studied with those presented in Table 2, the type of binder used, additives, curing time, and the original *R*^2^ of each mixture related to the porosity/binder index (original). The soils used for the analysis have been previously studied in the literature, and they are mainly poorly graded sands and sedimentary silt. The additives used are glass waste, in powder or ground.

It is specified that all the mixes presented in Table 3 will be used to relate the new index with the mechanical strength (unconfined compression and/or split tensile), except the last mix. The stiffness (*G_o_*) and durability against wetting–drying cycles (accumulated loss of mass—*ALM*) of Osorio, Porto Alegre, and Rio Pardo Sand–carbide lime (*CL*)–glass powder compacted blends will be used to relate the new index. The last one, sand–cement (Table 3), will be used to relate the new porosity–water/binder index (*ηC_w_*/*B_iv_*).

### 3.1. Application of the Porosity–Water/Binder Index to Predict the Strength of Compacted Blends

**Figure 1:**
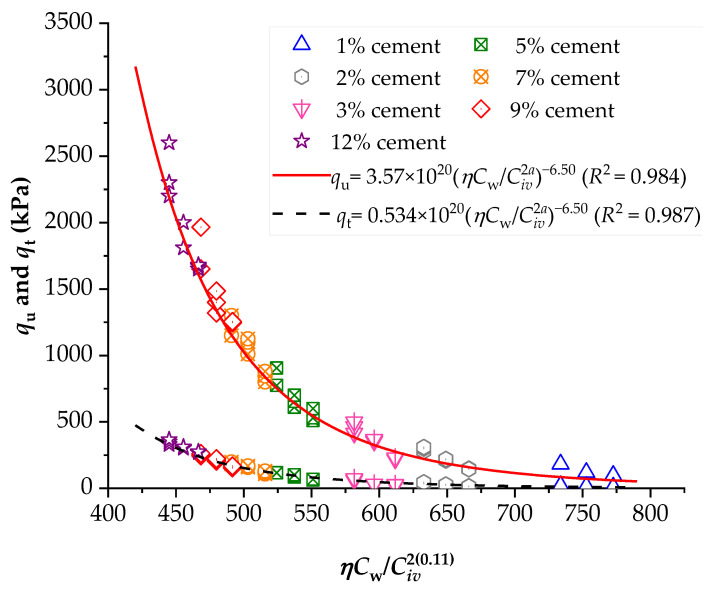
Influence of the porosity–water/binder index on the unconfined compressive and split tensile strengths of Brazilian Osorio sand–cement compacted blends [24].

**Figure 2:**
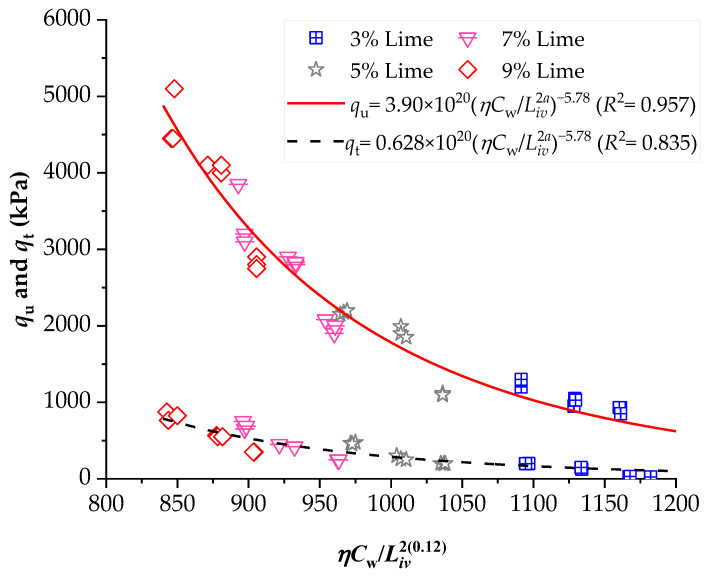
Influence of the porosity–water/binder index on the unconfined compressive and split tensile strengths of hydrated lime–soil compacted blends [25].

**Figure 3:**
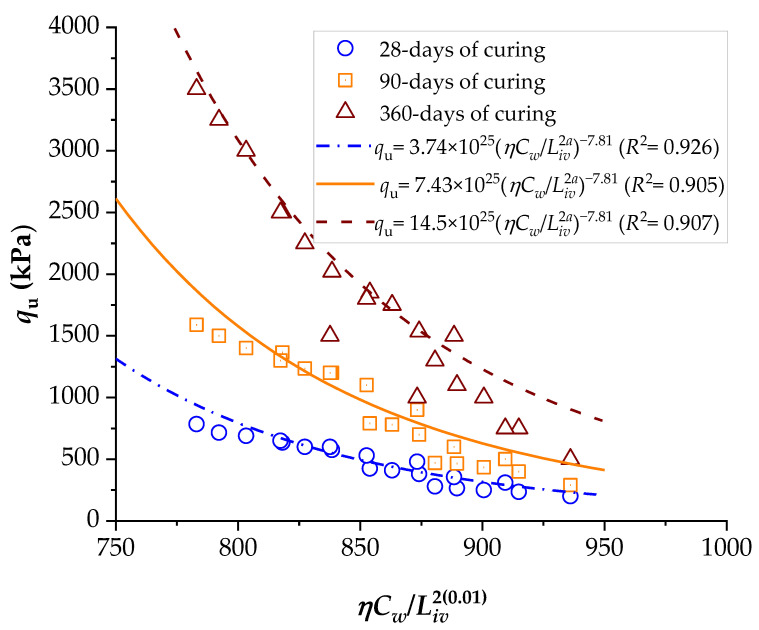
Influence of the porosity–water/binder index on the unconfined compressive strength of silty soil–hydrated lime compacted blends, considering 28, 90, and 360 days of curing [26].

**Figure 4:**
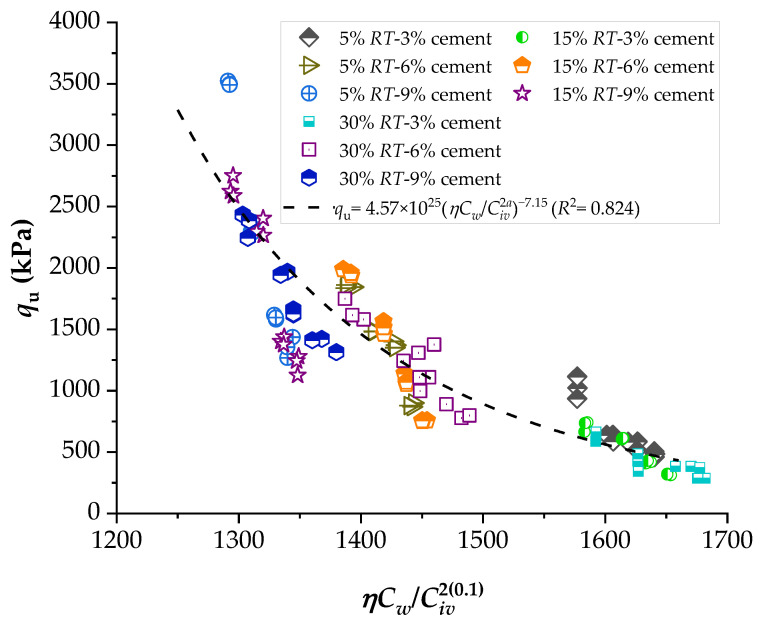
Influence of the porosity–water/binder index on the unconfined compressive strength of silty soil–cement–roof tile waste compacted blends, considering 28 days of curing [27].

**Figure 5:**
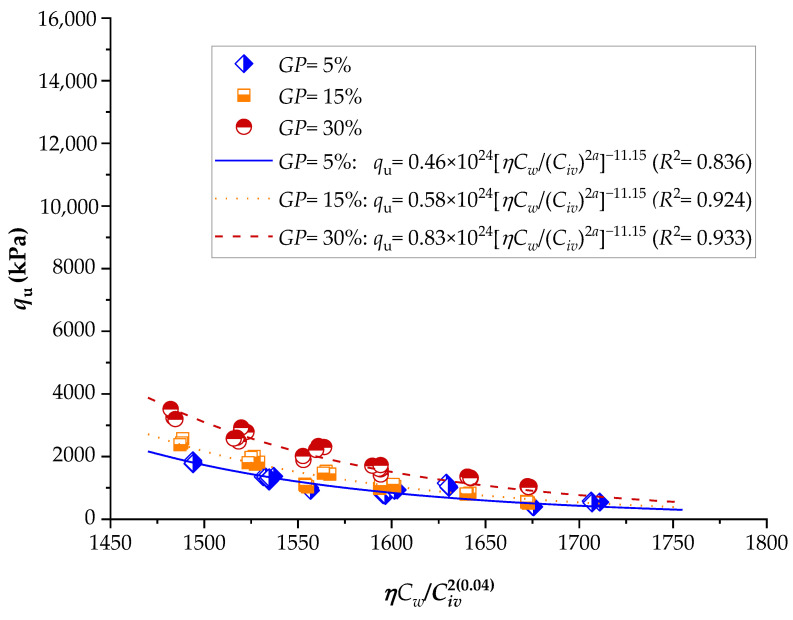
Influence of the porosity–water/binder index on the unconfined compressive strength of silty soil–cement–glass powder compacted blends, considering 7 days of curing [17].

**Figure 6:**
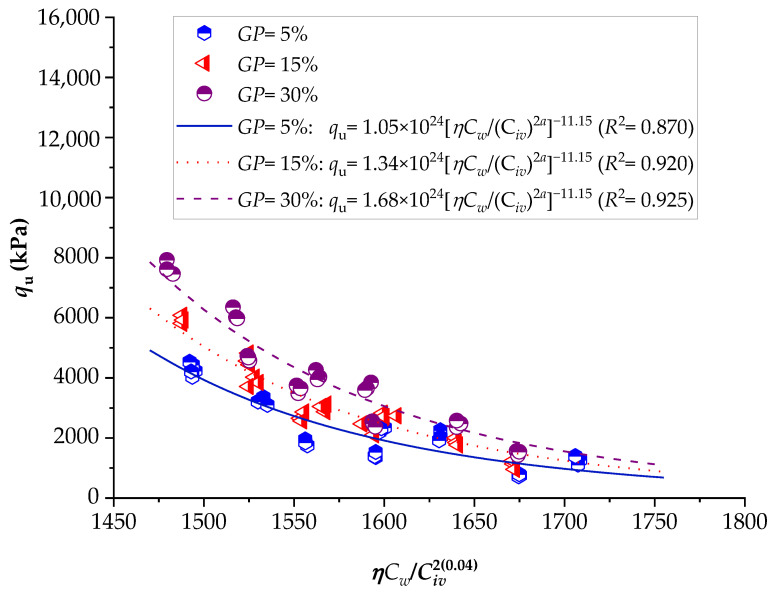
Influence of the porosity–water/binder index on the unconfined compressive strength of silty soil–cement–glass powder compacted blends, considering 28 days of curing [17].

**Figure 7:**
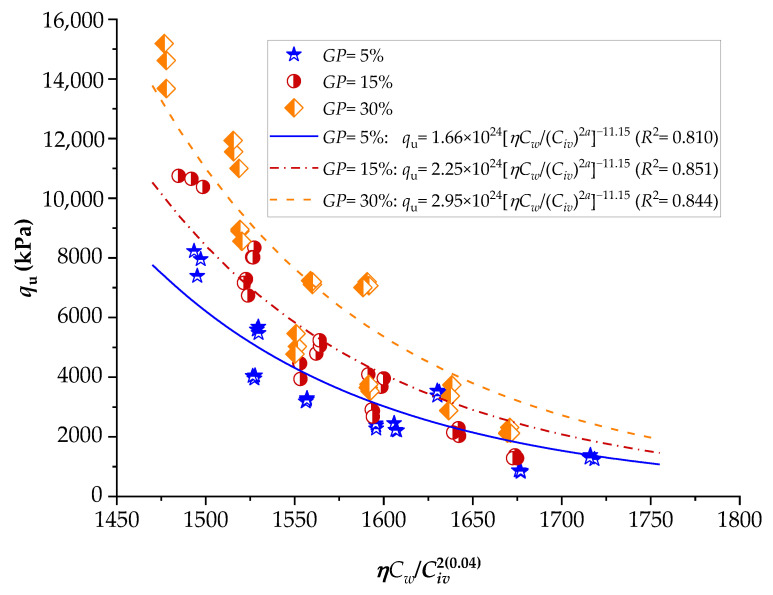
Influence of the porosity–water/binder index on the unconfined compressive strength of silty soil–cement–glass powder compacted blends, considering 90 days of curing [17].

**Figure 8:**
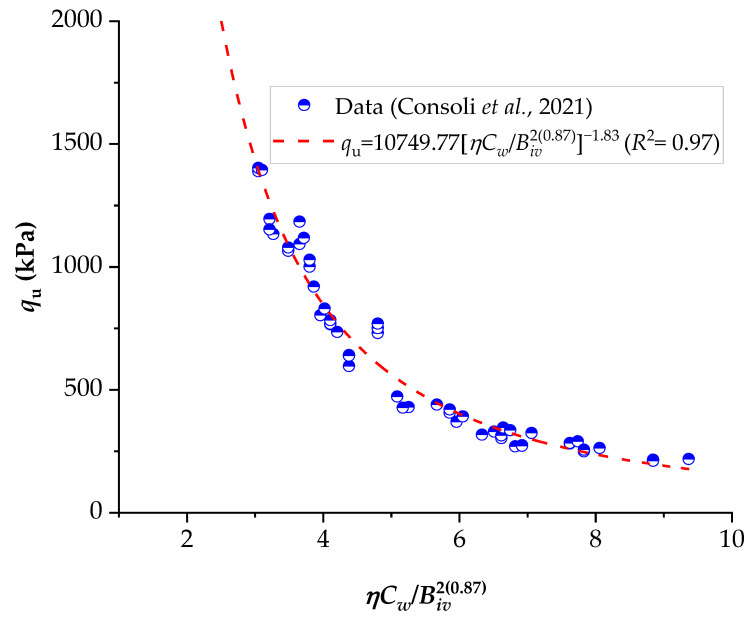
Influence of the porosity–water/binder index on the unconfined compressive strength of Osorio sand–carbide lime–glass powder compacted blends [13].

**Figure 9:**
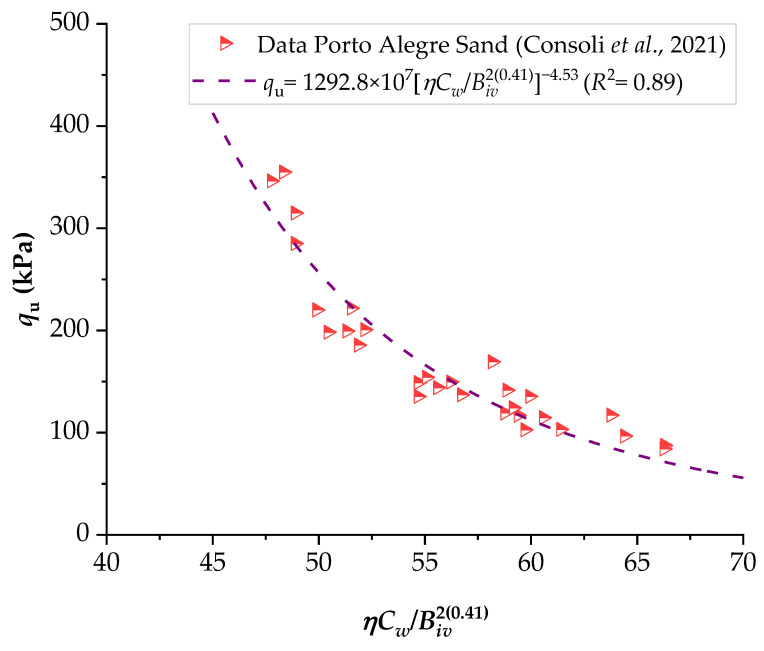
Influence of the porosity–water/binder index on the unconfined compressive strength of Porto Alegre sand–carbide lime–glass powder compacted blends [13].

**Figure 10:**
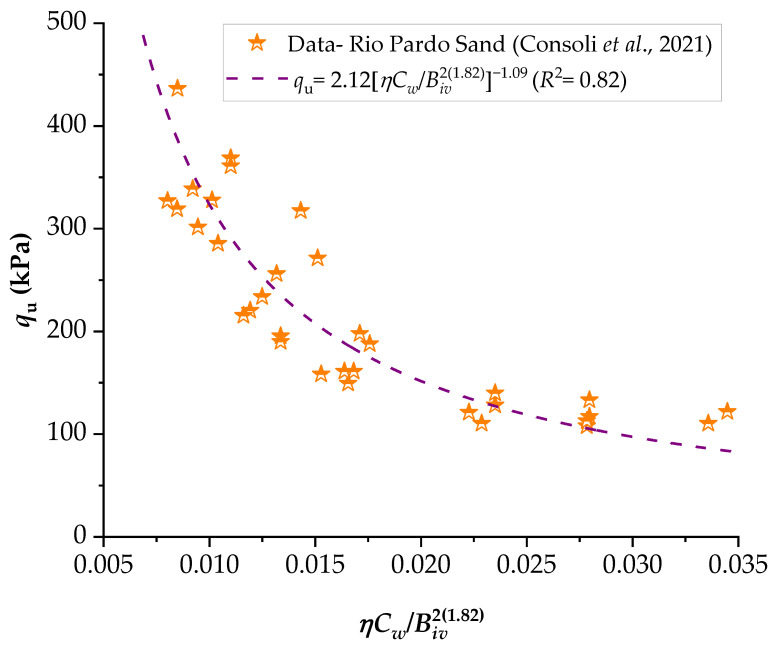
Influence of the porosity–water/binder index on the unconfined compressive strength of Rio Pardo sand–carbide lime–glass powder compacted blends [13].

To demonstrate the efficiency of this new index, the strengths of several stabilized soils were compared based on the *ηC_w_*/*B_iv_* index. The details of the stabilized soils are listed in Table 2 and Table 3. Figure 1, Figure 2, Figure 3, Figure 4, Figure 5, Figure 6, Figure 7, Figure 8, Figure 9 and Figure 10 show the potential fitting (based on Equation (6)) of strength in correlation with the *ηC_w_*/*B_iv_* index for sand–cement blends [24], silt–lime blends that were cured for 28 days (as presented by Consoli’s original study [25]), silty soil–lime compacted blends that were cured for up to one year [26], silt–cement–roof tile waste blends [26], silty soil–cement–glass powder blends [17], and various sand soils–CL–glass powder compacted blends [13].

From Figure 1, Figure 2, Figure 3, Figure 4, Figure 5, Figure 6, Figure 7, Figure 8, Figure 9 and Figure 10, the authors observed that, by using the exponent ‘*a*’, the fitting curve could be better adjusted to regulate the adjustment curve to the results. The equations presented superior coefficient of determination (*R*^2^) values between 0.81 and 0.99. The coefficients of determination are close to those calculated in the original research, in which the *η*/*B_iv_* parameter was used (Table 3). Moreover, Figure 1, Figure 2, Figure 3, Figure 4, Figure 5, Figure 6, Figure 7, Figure 8, Figure 9 and Figure 10 show that considering the amount of water added is appropriate for estimating the strength of the compacted mixes. In this respect, the newly proposed relationship can be used to predict the strength response of artificially cemented soils that have a broad range of porosities and binder contents, including the requirements of geotechnical field applications. In addition, Figure 1, Figure 2, Figure 3, Figure 4, Figure 5, Figure 6, Figure 7, Figure 8, Figure 9 and Figure 10 also show that the correlation between the volumes of voids–water and cement is more appropriate for presenting strength results, considering that, for a given variation in the volume of voids–water, a proportional variation in the volume of cement would balance the loss and gain in strength. To correct the disproportion (between the volume of voids and binder content) and maintain the dimensionless variables, the porosity was plotted as a function of the volumetric binder content adjusted by an exponent (generally close to *a* = 0.1, and, in some instances, *a* = 0.01), which is a function of the type of soil and binder used. On the other hand, the exponent *x* (from Equation (1)) is close to 1.0 (i.e., *x* = 1.0) for frictional soils and can take values between 0.11 and 0.28 for fine-grained soils.

Another interesting aspect that was explicitly observed in Figure 1 and Figure 2 is that, in soil–cement and lime–soil mixtures, by including the *C_w_* parameter, the tensile/compression index is independent of the degree of compaction, amount of binder, and amount of water. In the case of the soil–cement mixture, the tensile/compression index was calculated to be 0.15 (Figure 1), and, for the lime–soil mixture, it was 0.16 (Figure 2), which is similar to the values measured in the original studies [24,25]. In Figure 2, the coefficient of determination was calculated as 0.835 for the split tensile strength. Figure 7 shows that the *R*^2^ value in the modified fitting result is very different from the result of fitting in Baldovino’s study. The lower *R*^2^ observed in applying the new index to a specific soil may be attributed to inherent soil properties, potential interactions with unaccounted factors, or data variability. Further analysis is needed to assess soil characteristics, consider additional parameters, and address the limitations of the new index for more accurate predictions. In the case of this study, it is important to consider that stabilized soil systems are inherently complex and may be subject to natural and experimental variations. Furthermore, the introduction of a new parameter, such as the porosity–water/binder ratio, may require more detailed and refined tuning as it is further developed and better understood with future experiments. An *R*^2^ below 0.9 may suggest the possibility of other unknown or unaccounted-for factors in the model that influence the results, which is not in the scope of this research.

The porosity/cement index allows for predicting the strength of cemented soils by adjusting it for the specific soil of interest, making it applicable as a rational dosage methodology. For a more accurate fit, an exponent “*a*” is utilized, which depends on the soil type and the materials used in stabilization. In the assessment of the *q_t_* value for three distinct soils improved through the addition of Portland cement, Consoli et al. [28] observed that the volumetric content of the cementing agent is adjusted by a distinct exponent based on the soil type. For Osorio sand, the use of the exponent was unnecessary (*a* = 1), while, for the mixtures of cement–silty sand from Porto and clayey sand from Botucatu-cement, the exponents were 0.21 and 0.28, respectively.

In a comprehensive examination of improving the performance of cement-mixed soils, multiple studies have been conducted. These studies (e.g., [29,30,31,32,33]) collectively explore the impact of various factors, including water-to-cement ratio, cement content, porosity, and their combined influence on the strength of cement-mixed soils. A novel index, the combined material ratio, has been introduced for strength analysis, providing a clearer understanding of the interplay between water-to-cement ratio, dry density, and strength mobilization [33]. Additionally, research has proposed a blended volume ratio as an effective index for evaluating cement-mixed soil strength, considering the concurrent effects of porosity, cement amount, and water amount. Moreover, the study suggests an empirical equation based on the blended volume ratio to predict cement-mixed soil strength, which has been validated against various datasets, demonstrating its practical applicability [32]. Another investigation [31] explores the mechanical behavior of cemented sands with a focus on heterogeneity properties, utilizing the discrete element method (DEM) model and Weibull statistics to analyze their influence. Furthermore, the study delves into the enhanced mechanical properties and durability of coal gangue-reinforced cement–soil mixtures, highlighting substantial strength improvements and anti-corrosion benefits resulting from coal gangue incorporation. Lastly, research elucidates a generalized strength prediction equation for cement-stabilized clayey soils [34], emphasizing the pivotal role of clay minerals, water content, and cement content in strength development. Additionally, an experimental study introduces the concept of the composite, composed of steel slag, cement, and metakaolin, as an effective means to enhance soil strength, with the proposed free water content serving as a valuable parameter for characterizing unconfined compression strength in binder-stabilized soils [30]. These findings collectively contribute to advancing our understanding of optimizing soil performance through various material ratios and indices, with implications for geotechnical engineering and infrastructure development.

### 3.2. Application of the Porosity–Water/Binder Index to Predict the Stiffness of Compacted Blends

**Figure 11:**
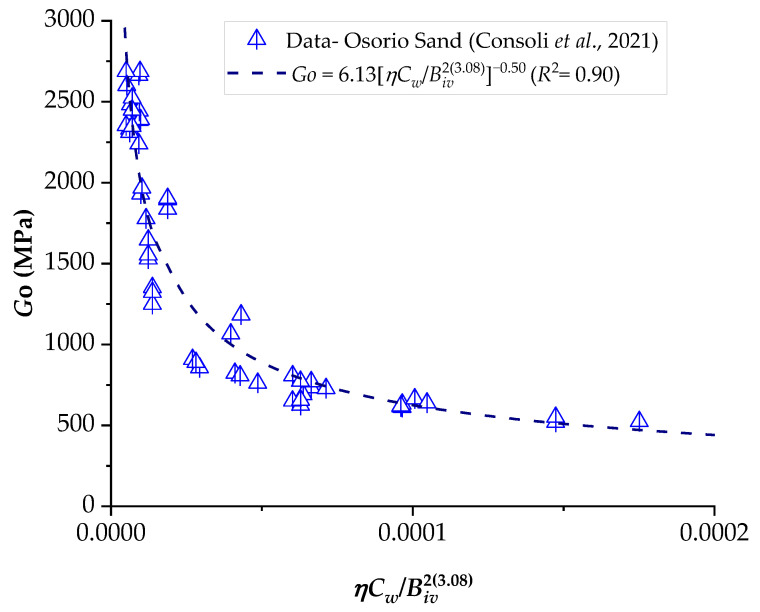
Influence of the porosity–water/binder index on the stiffness of Osorio sand–carbide lime–glass powder compacted blends.

**Figure 12:**
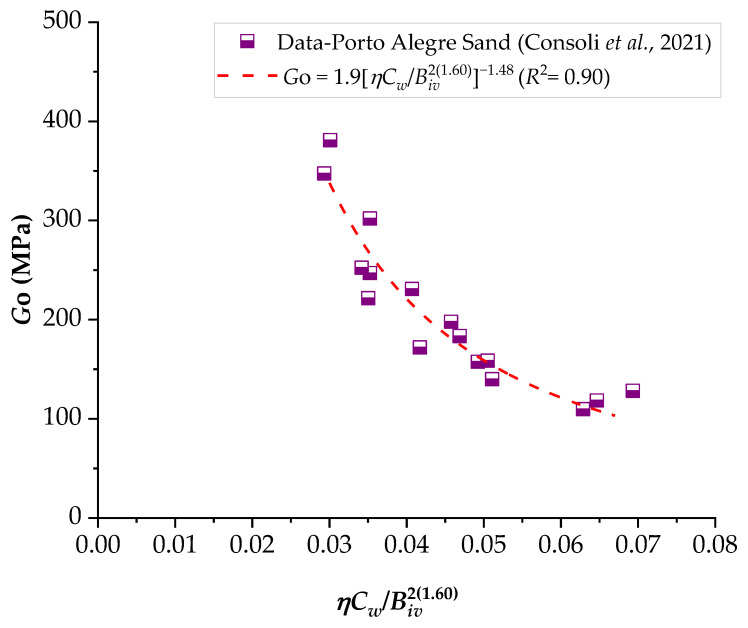
Influence of the porosity–water/binder index on the stiffness of Porto Alegre sand–carbide lime–glass powder compacted blends.

**Figure 13:**
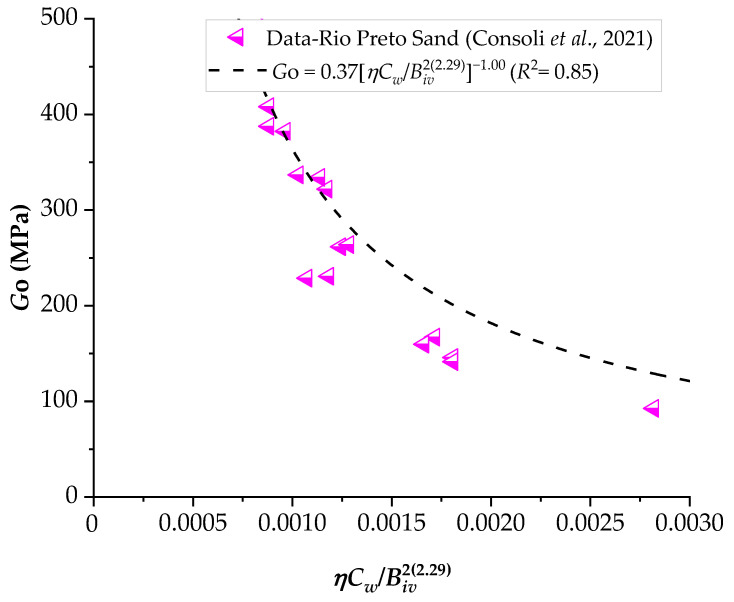
Influence of the porosity–water/binder index on the stiffness of Rio Preto sand–carbide lime–glass powder compacted blends.

The results of the ultrasonic pulse test were correlated with the *ηC_w_*/*B_iv_* index, analogous to what was presented in the results of unconfined compression, as shown in Figure 11, Figure 12 and Figure 13. It is noted that the behavior of the initial stiffness directly referred to that observed in the mechanical strength results, where the reduction of the porosity–cementing agent ratio led to an increase in unconfined compression. In general, higher initial moisture content resulted in higher values of *G_o_*.

A study conducted by Consoli et al. [35] observed that the porosity/cement index is suitable for assessing the initial stiffness (*G_o_*) and the *q_u_* value of soil–cement mixtures. This study considered two sandy soils with different levels of Portland cement (2–7%) and porosities. According to the authors, the reduction in porosity led to an increase in *q_u_* due to the higher number of grain contacts. For the same porosity, well-graded sand exhibited higher resistance values compared to uniform sand, suggesting a significant influence of the soil’s particle size distribution on its mechanical behavior. Furthermore, for both soils, *q_u_* and initial stiffness *G_o_* increased nearly linearly with the addition of cement content.

Consoli et al. [36] investigated the influence of different types of Portland cement, binder contents (3–9%), densities, curing times, and the porosity/cement *η*/*C_iv_* index on the *q_u_* of sand–cement mixtures. In this study, the *η*/*C_iv_* index was found to be suitable for evaluating the *q_u_* of mixtures for all cement types and curing periods. Additionally, a unique equation (relating *η*/*C_iv_*, *q_u_*, and curing time) was derived, applicable to various cement types. Consequently, for a specific cement type and desired curing period, it becomes possible to determine the appropriate cement content and porosity for the mixture to achieve the defined project strength.

In general, Portland cement contents ranging from 1 to 17% have been employed, and it is evident that the strength and stiffness of sand–cement mixtures are a function of dosage, porosity, and volumetric cement content.

### 3.3. Application of the Porosity–Water/Binder Index to Predict the Durability against Wetting–Drying Cycles (Accumulated Loss of Mass, ALM)

**Figure 14:**
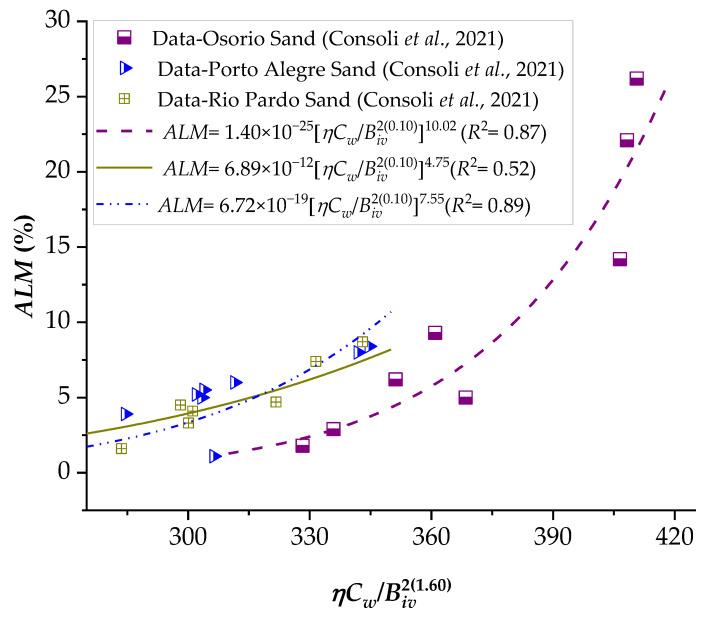
Influence of the porosity–water/binder index on the accumulated loss of mass (*ALM*) of various sandy soils–carbide lime–glass powder compacted blends.

**Figure 15:**
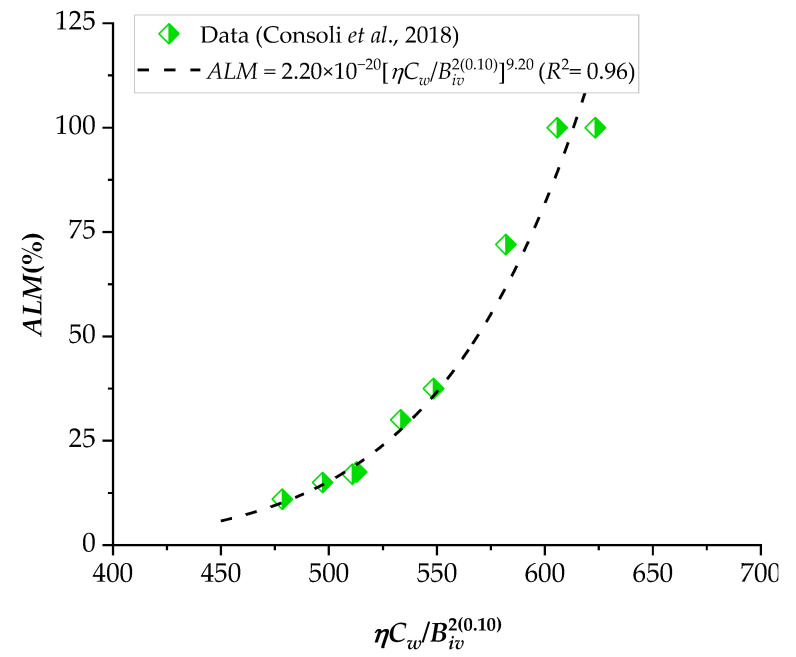
Influence of the porosity–water/binder index on the accumulated loss of mass (*ALM*) of Osorio sand–Portland cement mixes.

The addition of cement in sandy soils promotes improvements in strength, stiffness, and durability, as verified in several studies in the literature [13,21,37]. The *ALM* results of the sand–binder in 12 wetting–drying cycles were correlated with the *ηC_w_*/*B_iv_*, as presented in Figure 14 and Figure 15 for various silica sands combined with CL and ground glass. In line with the observations in the strength and stiffness results, a lower porosity–cementing agent ratio leads to a reduction in *ALM*, as explained by Consoli et al. [13]. The reduction in porosity maximizes particle contacts, friction mobilization, and interlocking, directly contributing to mechanical resistance against brush abrasion and wetting–drying cycles. According to the minimum durability requirements established by the Portland Cement Association [38], the maximum *ALM* after 12 cycles of wetting and drying is 14% for non-plastic fine sands. Thus, considering the results in Figure 14 and Figure 15, Osorio sand does not meet this requirement, but it establishes an excellent correlation with the proposed index. In addition, Rio Pardo sand fit is poorly attributed, perhaps due to inherent soil properties, potential interactions with unaccounted factors, or data variability. To find the factors that cause there to be no good correlation between *ALM* and porosity–water/index, future studies are required in which the microstructure of the mixtures is analyzed.

## 4. Discussion

The general solution based on Equation (6) allows the application of the index for the prediction of the mechanical behavior (e.g., strength, stiffness, durability, and resilient modulus) of any soil–cement mixture, including problematic soils such as tropical, expansive, and collapsible soils. The high coefficients of determination indicate that the proposed index can indeed be implemented to design and analyze soil–cement projects, encompassing a wide range of soils and both traditional and unconventional cementing materials.

A field engineer can follow a systematic approach to applying the presented concepts in practice. First, thoroughly characterize the properties of the soil, including its composition and initial strength. Then, calculate the predicted mechanical behavior using the proposed index. Select an appropriate cementitious binder based on the calculated values and determine the optimal mixture proportions. Conduct laboratory testing to validate the predictions and adjust the mixture if necessary. Implement the optimized mixture on-site, ensuring proper compaction and curing procedures. Continuously monitor the performance of the stabilized soil–cement mixture through field inspections and testing, comparing the actual behavior with the predicted values. By following these steps, engineers can effectively utilize the presented concepts in real-world scenarios, even when dealing with challenging soil conditions. 

In engineering infrastructure development, a pivotal consideration revolves around the load-bearing capacity of the underlying soil mass, which will provide essential support to the envisaged structure. In certain instances, geotechnical engineers are confronted with soils exhibiting a diminished load-bearing capacity, a discernment facilitated by meticulous geotechnical assessments [39]. These assessments offer insights into distinct regions, strata, or soil conglomerates characterized by adverse resistance and deformability attributes, thereby impacting the proposed ground intervention [40].

In response to these challenges, three discrete strategies emerge as prospective avenues for project implementation: firstly, the substitution of the unsuitable soil stratum with materials boasting superior mechanical properties; secondly, the adaptation of the project design to harmonize with the distinct characteristics of the local soil; and, thirdly, the deployment of innovative soil enhancement techniques aimed at the deliberate modification of soil properties. Each strategy underscores the interplay between engineering acumen, geological intricacies, and the pursuit of optimal and sustainable solutions in geotechnical practice [41].

The resultant material from the soil–cement technique exhibits distinctions from conventional concrete across several facets. One of the primary disparities lies in the fact that concrete encompasses a sufficient quantity of paste (comprising cement and water) to thoroughly coat the surface of aggregates within the mixture and occupy void spaces. Conversely, in soil–cement blends, the paste quantity falls short in enveloping the entire soil particle surface and filling the voids present. This disparity culminates in a cementitious matrix binding together nodules of non-cemented aggregates [42].

Although the majority of the studies presented to demonstrate the effectiveness of a novel index in controlling mechanical and durability properties focus on stabilized mixtures up to 7 days of curing, there is considerable interest in the long-term interaction between soil and binder, extending, for example, to 360 days. This paper provides evidence that the new index governs long-term mechanical properties. However, more extensive investigations are required to validate whether other binders do not interact favorably with the porosity–water/binder relationship. Thus, the critical role of extended curing times in soil stabilization research is emphasized, particularly in the context of non-standard composites involving soils of varying hydraulic binder contents. While much of the existing literature predominantly focuses on short curing durations, typically up to 7 days, recent studies have underscored the substantial benefits of longer curing periods, extending to several months or even a year. This growing body of evidence highlights the dynamic nature of soil–binder interactions and the evolving material characteristics over time. Given the increasing relevance of sustainable construction practices and the expanding utilization of soil-stabilized materials, a comprehensive exploration of extended curing effects is imperative to enhance our understanding and improve engineering practices.

Field engineers can use the *ηC_w_*/*B_iv_* index to select the best option that satisfies their requirements. This can be achieved by either decreasing the porosity (which can be interpreted as increasing soil compaction) and amount of binder or increasing the porosity and binder content. Furthermore, according to da Rocha et al. [43], an increase in soil compaction energy is less environmentally friendly than an increase in the chemical stabilizer content. Consoli et al. [44] also applied the presented index and studied a wide range of materials, including problematic soils, fine-grained soils, and mine tailings; the authors realized that stabilized materials with an initial shear modulus higher than 2.500 MPa were not a cause of concern in terms of durability when subjected to wet–dry cycles. In this context, the *η*/*B_iv_* index can be considered as a reliable tool for soil stabilization and dosing methodology to guarantee the desired performance of the selected blends. 

## 5. Conclusions

In this paper, a new interpretation of the application of the porosity/binder relationship is presented to represent the evolution of the strength of artificially cemented soils. To verify the proposed index, five soils that were previously studied in the literature were used to demonstrate the efficacy of the new porosity–water/binder index. Thus, the following conclusions were drawn:

- This study introduces a novel perspective on the application of the porosity/binder relationship, underscoring its potential in delineating the strength evolution of artificially cemented soils.
- This research establishes the porosity–water/binder index as a multifaceted instrument, adept at assessing the strengths of varied artificially cemented soils, highlighting its pivotal role in subsequent geotechnical research.
- These findings corroborate the consistent impact of water content on the *q_t_*/*q_u_* values across different soil blends, bolstering the credibility of antecedent research insights.
- The efficacy of the porosity–water/binder ratio in addressing a spectrum of soil types, especially those inherently challenging from a geotechnical standpoint, accentuates its indispensability in a multitude of stabilization methodologies.
- To further this avenue of investigation, it is imperative to explore the intricacies of this ratio’s influence on the durability and resilience of cemented soils.
- The expansive applicability scope of the porosity–water/binder index, spanning an array of geological contexts, manifests its prospective capacity to instigate transformative shifts in modern geotechnical engineering practices.
- The analysis of the influence of water content on the ratio of splitting tensile strength to compressive strength (*q_t_*/*q_u_*) for stabilized soils revealed consistent results across soil–cement and lime–soil blends. The *q_t_*/*q_u_* values of 0.15 and 0.16 for soil–cement and lime–soil blends, respectively, aligned well with the values reported in previous studies.

## Figures and Tables

**Table 1:** Values of the parameters *a* and 1/*a* for the soils stabilized with different binders and residues.

Mix Design	Parameter *a*	Parameter 1/*a*	Theoretical *a*	*p*/*a*	Reference
Silty soil–cement–recycled glass powder	3.87	0.20	0.26	0.77/*β*	[17]
Silty soil–cement–recycled glass powder	3.92	0.22	0.26	0.86/*β*	[18]
Cement-treated soil	3.30	0.44	0.30	1.45/*β*	[19]
Cement-treated soil	3.30	0.44	0.30	1.45/*β*	[19]
Silty soil–lime	4.39	0.20	0.23	0.88/*β*	[20]
Cement-treated soil–ground tile waste	4.47	0.28	0.22	1.25/*β*	[21]
Osorio sand–lime–glass powder	3.60	0.28	0.28	1.00/*β*	[13]
Porto Alegre sand–lime–glass powder	3.60	0.28	0.28	1.00/*β*	[13]
Rio Pardo sand–lime–glass powder	3.60	0.28	0.28	1.00/*β*	[13]

**Table 2:** Geotechnical properties of the stabilized soils studied.

Soil Detail	Sand (%)	Silt (%)	Clay (%)	Limit Liquid (%)	Plasticity Index (%)	USCS Classification	Specific Gravity	Mainly Minerals	Reference
Osorio sand	100	-	-	-	-	SP	2.65	Quartz	Consoli et al. [13,24]
Porto Alegre sand	100	-	-	-	-	SP	2.65	Quartz	Consoli et al. [13]
Rio Pardo sand	100	-	-	-	-	SP	2.68	Quartz	Consoli et al. [13]
Silt–lime	1.5	65.5	33	39	4	ML	2.64	Kaolinite	Consoli et al. [25]
Porto Alegre sandy clay	53.7	42	4.3	24	9	CL	2.81	Kaolinite	Consoli et al. [26]
Guabirotuba silt	33.4	57.6	9.3	53.1	21.3	MH	2.71	Kaolinite	Moireira et al. [27]
Southern Brazil soil	35	60	5	50.8	14.9	MH	2.62	Kaolinite and quartz	Baldovino et al. [17]

**Table 3:** Details of the stabilized soils to apply the efficiency of the proposed porosity–water/binder index.

Reference	Type of Mix	Amount of the Additives (%)	% Cement or Lime	Curing Days	Water Content *w* (%)	Original *R*^2^
[24]	Osorio sand–cement	-	1, 2, 3, 5, 7, 9 and 12	7	10	0.97–0.98
[25]	Silt–lime	-	3, 5, 7 and 9	28	20	0.82–0.95
[26]	Silty soil–lime	-	3, 5, 7 and 9	28, 90 and 360	23	0.89–0.96
[27]	Soil–roof tile–cement	5, 15 and 30	3, 6 and 9	28	23	0.97–0.98
[17]	Soil–glass powder–cement	5, 15 and 30	3, 6 and 9	7, 28 and 90	26	0.88–0.99
[13]	Osorio sand–carbide lime–glass powder *	10 and 20	3, 5 and 7	7	10	0.86 **
[13]	Porto Alegre sand–carbide lime–glass powder *	10 and 20	3, 5 and 7	7	10	0.64 **
[13]	Rio Pardo sand–carbide lime–glass powder *	10 and 20	3, 5 and 7	7	10	0.77 **
[21]	Sand–cement ***	-	3, 6, and 9	7	10	-

* For *G_o_* analysis; ** valid only for *q_u_* analysis; *** only for durability analysis.

## Data Availability

No further data is available.

## References

[1] Consoli N.C., Scheuermann Filho H.C., Leon H.B., da Silva Carretta M., Corte M.B., Cordeiro R.E., Caballero R.D., Lourenço D.E. (2021). General Relationships Controlling Loss of Mass, Stiffness and Strength of Sustainable Binders Amended Sand. Transp. Geotech..

[2] Ekinci A., Hanafi M., Aydin E. (2020). Strength, Stiffness, and Microstructure of Wood-Ash Stabilized Marine Clay. Minerals.

[3] Hanafi M., Ekinci A., Aydin E. (2020). Triple-Binder-Stabilized Marine Deposit Clay for Better Sustainability. Sustainability.

[4] Henzinger C., Schuhmacher S.A., Festugato L. (2018). Applicability of the Porosity/Binder Index to Nonhomogeneous Mixtures of Fine-Grained Soil with Lignite Fly Ash. J. Mater. Civ. Eng..

[5] Ermolovich E.A., Ivannikov A.L., Khayrutdinov M.M., Kongar-Syuryun C.B., Tyulyaeva Y.S. (2022). Creation of a Nanomodified Backfill Based on the Waste from Enrichment of Water-Soluble Ores. Materials.

[6] Kanty P., Rybak J., Stefaniuk D. (2017). Some Remarks on Practical Aspects of Laboratory Testing of Deep Soil Mixing Composites Achieved in Organic Soils. IOP Conf. Ser. Mater. Sci. Eng..

[7] Consoli N.C., Foppa D., Festugato L., Heineck K.S. (2007). Key Parameters for Strength Control of Artificially Cemented Soils. J. Geotech. Geoenviron. Eng..

[8] Consoli N.C., da Silva Lopes L., Foppa D., Heineck K.S. (2009). Key Parameters Dictating Strength of Lime/Cement-Treated Soils. Proc. Inst. Civ. Eng.-Geotech. Eng..

[9] Consoli N.C., Bellaver Corte M., Festugato L. (2012). Key Parameter for Tensile and Compressive Strength of Fibre-Reinforced Soil–Lime Mixtures. Geosynth. Int..

[10] Diambra A., Ibraim E., Peccin A., Consoli N.C., Festugato L. (2017). Theoretical Derivation of Artificially Cemented Granular Soil Strength. J. Geotech. Geoenviron. Eng..

[11] Diambra A., Festugato L., Ibraim E., Peccin da Silva A., Consoli N.C. (2018). Modelling Tensile/Compressive Strength Ratio of Artificially Cemented Clean Sand. Soils Found..

[12] Festugato L., Peccin da Silva A., Diambra A., Consoli N.C., Ibraim E. (2018). Modelling Tensile/Compressive Strength Ratio of Fibre Reinforced Cemented Soils. Geotext. Geomembr..

[13] Consoli N.C., da Silva Carretta M., Festugato L., Leon H.B., Tomasi L.F., Heineck K.S. (2021). Ground Waste Glass–Carbide Lime as a Sustainable Binder Stabilising Three Different Silica Sands. Géotechnique.

[14] Ekinci A., Abki A., Mirzababaei M. (2022). Parameters Controlling Strength, Stiffness and Durability of a Fibre-Reinforced Clay. Int. J. Geosynth. Ground Eng..

[15] Hanafi M., Ekinci A., Aydin E. (2022). Engineering and Microstructural Properties of Alluvium Clay Stabilized with Portland Cement and Coal Bottom Ash for Sustainable Future. KSCE J. Civ. Eng..

[16] Lade P.V., Trads N. (2014). The Role of Cementation in the Behaviour of Cemented Soils. Geotech. Res..

[17] Baldovino J.d.J., dos Santos Izzo R.L., da Silva É.R., Lundgren Rose J. (2020). Sustainable Use of Recycled-Glass Powder in Soil Stabilization. J. Mater. Civ. Eng..

[18] Baldovino J., Izzo R., Rose J.L., Avanci M.A. (2020). Geopolymers Based on Recycled Glass Powder for Soil Stabilization. Geotech. Geol. Eng..

[19] Baldovino J.d.J.A., Izzo R.L.d.S., Feltrim F., da Silva É.R. (2020). Experimental Study on Guabirotuba’s Soil Stabilization Using Extreme Molding Conditions. Geotech. Geol. Eng..

[20] Baldovino J.A., Moreira E.B., Izzo R.L.d.S., Rose J.L. (2018). Empirical Relationships with Unconfined Compressive Strength and Split Tensile Strength for the Long Term of a Lime-Treated Silty Soil. J. Mater. Civ. Eng..

[21] Consoli N.C., Tomasi L.F. (2018). The Impact of Dry Unit Weight and Cement Content on the Durability of Sand–Cement Blends. Proc. Inst. Civ. Eng.-Ground Improv..

[22] Rios S., Viana da Fonseca A., Baudet B.A. (2012). Effect of the Porosity/Cement Ratio on the Compression of Cemented Soil. J. Geotech. Geoenviron. Eng..

[23] Horpibulsuk S., Miura N., Nagaraj T.S. (2003). Assessment of Strength Development in Cement-Admixed High Water Content Clays with Abrams’ Law as a Basis. Géotechnique.

[24] Consoli N.C., Cruz R.C., Floss M.F., Festugato L. (2010). Parameters Controlling Tensile and Compressive Strength of Artificially Cemented Sand. J. Geotech. Geoenviron. Eng..

[25] Consoli N.C., Dalla Rosa Johann A., Gauer E.A., Dos Santos V.R., Moretto R.L., Corte M.B. (2012). Key Parameters for Tensile and Compressive Strength of Silt-Lime Mixtures. Geotech. Lett..

[26] Consoli N.C., Prietto P.D.M., da Silva Lopes L., Winter D. (2014). Control Factors for the Long Term Compressive Strength of Lime Treated Sandy Clay Soil. Transp. Geotech..

[27] Moreira E.B., Baldovino J.A., Rose J.L., Luis dos Santos Izzo R. (2019). Effects of Porosity, Dry Unit Weight, Cement Content and Void/Cement Ratio on Unconfined Compressive Strength of Roof Tile Waste-Silty Soil Mixtures. J. Rock Mech. Geotech. Eng..

[28] Consoli N.C., da Fonseca A.V., Cruz R.C., Silva S.R. (2011). Voids/Cement Ratio Controlling Tensile Strength of Cement-Treated Soils. J. Geotech. Geoenviron. Eng..

[29] Liu L., Zhou A., Deng Y., Cui Y., Yu Z., Yu C. (2019). Strength Performance of Cement/Slag-Based Stabilized Soft Clays. Constr. Build. Mater..

[30] Long G., Li L., Li W., Ma K., Dong W., Bai C., Zhou J.L. (2019). Enhanced Mechanical Properties and Durability of Coal Gangue Reinforced Cement-Soil Mixture for Foundation Treatments. J Clean. Prod..

[31] Wu M., Huang R., Wang J. (2021). DEM Simulations of Cemented Sands with a Statistical Representation of Micro-Bond Parameters. Powder Technol..

[32] Pham T.A., Kyokawa H., Koseki J., Dias D. (2023). A New Index for the Strength Analysis and Prediction of Cement-Mixed Soils. Eur. J. Environ. Civ. Eng..

[33] Pham T.A., Koseki J., Dias D. (2021). Optimum Material Ratio for Improving the Performance of Cement-Mixed Soils. Transp. Geotech..

[34] Sukmak G., Sukmak P., Horpibulsuk S., Arulrajah A., Horpibulsuk J. (2023). Generalized Strength Prediction Equation for Cement Stabilized Clayey Soils. Appl. Clay. Sci..

[35] Consoli N.C., da Fonseca A.V., Silva S.R., Cruz R.C., Fonini A. (2012). Parameters Controlling Stiffness and Strength of Artificially Cemented Soils. Geotechnique.

[36] Consoli N.C., Festugato L., da Rocha C.G., Cruz R.C. (2013). Key Parameters for Strength Control of Rammed Sand–Cement Mixtures: Influence of Types of Portland Cement. Constr. Build. Mater..

[37] Carvalho Queiróz L., Dias Miguel G., Jordi Bruschi G., Deluan Sampaio de Lima M. (2022). Macro–Micro Characterization of Green Stabilized Alkali-Activated Sand. Geotech. Geol. Eng..

[38] Portland Cement Association (2000). Soil-Cement Laboratory Handbook.

[39] Kluge K., Kirsch A., Budach C. (2022). Digitale Lehre in der Geotechnik: Aktueller Stand und weitere Entwicklungen. Geotechnik.

[40] Cui Z.-D. (2018). Land Subsidence Induced by the Engineering-Environmental Effect.

[41] Mickovski S.B. (2016). Why Is the Future Ready for Environmental Geotechnics?. Environ. Geotech..

[42] Kemeny J., Berman E., Bracke P., Brent W., Budhu M., Coleman A., Dempsey R., Frumkin J., Oxnam M., Przybylski L. GROW: A Digital Library for Geotechnical, Rock, and Water Aspects of Civil Engineering. Proceedings of the 2002 ASEE Annual Conference and Exposition: Vive L’ingenieur.

[43] da Rocha C.G., Passuello A., Consoli N.C., Quiñónez Samaniego R.A., Kanazawa N.M. (2016). Life Cycle Assessment for Soil Stabilization Dosages: A Study for the Paraguayan Chaco. J. Clean. Prod..

[44] Consoli N.C., Festugato L., Filho H.C.S., Miguel G.D., Neto A.T., Andreghetto D. (2020). Durability Assessment of Soil-Pozzolan-Lime Blends through Ultrasonic-Pulse Velocity Test. J. Mater. Civ. Eng..

